# Attention deficit and hiperactivity disorder: Age, sex and social environment as evaluable factors in children and teenagers

**DOI:** 10.1192/j.eurpsy.2021.559

**Published:** 2021-08-13

**Authors:** S.S. Sánchez Rus

**Affiliations:** Psychiatrist, Mental Health Unit, Ugc Jaén, University Hospital, Complejo Hospitalario Jaén, Jaén, Spain

**Keywords:** Hiperactivity, attention deficit, children and teenagers, ADHD

## Abstract

**Introduction:**

Attention Deficit and Hiperactivity Disorder (ADHD) is a neurobiological disorder, affecting executive functions and defined by hyperactivity, attention deficit and/or impulsivity symptoms. This neurodevelopmental disorder affect up to 7% of children. It is observed as a chronic pathology appearing on childhood with other comorbid diseases. Often, remarkable symptoms use to change with the age, however a real improvement is also related with other -external- factors, as social environment.

**Objectives:**

-To highlight variability of ADHD symptoms and complexity of available treatments in childhood. -To analize influence of personal and familiar factors, which affect to evolution of ADHD and the response to treatment.

**Methods:**

Comparative-study. Retrospective selection of 8 patients with treatment for ADHD and currently stable. A 12-months ADHD confirmed diagnosis in Child and Adolescent Mental Health Unit and follow-up after diagnosis. They are compared by aged-pairs (females and males) at the different development school-stages (preschool 3-6 years, primary school 7-12 years and adolescence 13-16 years). *Pairs of study: male-5 years/female-6 years; male-7 years/female-8 years; female-10 years/male-11 years; female-13 years/male-15 years

**Results:**

-Evolution of ADHD highlights the influence of age-factor about remarkable symptoms mainly (from hiperactivity to inattentiveness). -Comorbid disorders seems related with sex-factor (impulsivity-eating disorders on females and irritability-mood disorders on males). -Children social environment, specially family support, is an important external factor for all these patients (low self-steem or somatization disorders).

**Conclusions:**

1. ADHD as a chronic disorder whose evolution depends on the age, sex and social factors 2. Genetic component or familiar support are also considered as influencers factors 3.Multidisciplinary approach to objectives: motivation, organization and maturity 4. Treatment is consider according to side effects and comorbidity

